# 3,3-Dimethyl-10-(4-methoxy­phen­yl)-9-(4-nitro­phen­yl)-1,2,3,4,5,6,7,8,9,10-deca­hydro­acridine-1,8-dione^1^

**DOI:** 10.1107/S1600536808017212

**Published:** 2008-06-13

**Authors:** Chunbao Miao, Changsheng Yao, Shujiang Tu, Xiaoqiang Sun

**Affiliations:** a Jiangsu Provincial Key Laboratory of Fine Petrochemical Engineering, Jiangsu Polytechnic University, Changzhou 213164, People’s Republic of China; b School of Chemistry and Chemical Engineering, Xuzhou Normal University, Xuzhou 221116, People’s Republic of China

## Abstract

The title compound, C_28_H_28_N_2_O_5_, consists of a partially hydrogenated acridine ring system with two substituted phenyl substituents on the dihydro­pyridine ring which are both nearly perpendicular to the mean plane of the acridine unit [dihedral angles of 81.3 (1) and 89.6 (1)° between the central ring of acridine and the methoxyphenyl and nitrophenyl rings, respectively]. The dihydro­pyridine ring is almost planar, whereas both the outer unsymmetrical six-membered rings adopt half-chair conformations.

## Related literature

For related literature, see: Ganesh *et al.* (1998 ▶); Jang *et al.* (2005 ▶); Shanmugasundaram *et al.* (1996 ▶); Wang *et al.* (2003 ▶).
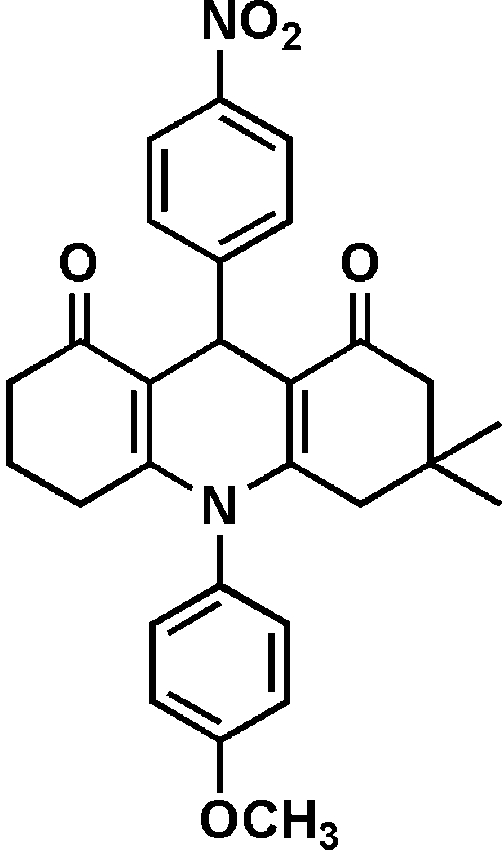


## Experimental

### 

#### Crystal data

C_28_H_28_N_2_O_5_
*M* *_r_* = 472.52
Monoclinic, 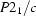

*a* = 12.463 (2) Å
*b* = 12.104 (2) Å
*c* = 16.408 (3) Å
β = 98.251 (5)°
*V* = 2449.6 (7) Å^3^
*Z* = 4
Mo *K*α radiation
μ = 0.09 mm^−1^
*T* = 293 (2) K
0.80 × 0.59 × 0.58 mm

#### Data collection

Rigaku Mercury diffractometer
Absorption correction: multi-scan (*CrystalClear*; Rigaku, 2001 ▶) *T*_min_ = 0.760, *T*_max_ = 0.950
23202 measured reflections
4466 independent reflections
3901 reflections with *I* > 2σ(*I*)
*R*_int_ = 0.030

#### Refinement

*R*[*F* ^2^ > 2σ(*F* ^2^)] = 0.054
*wR*(*F* ^2^) = 0.123
*S* = 1.17
4466 reflections
338 parameters
H-atom parameters constrained
Δρ_max_ = 0.15 e Å^−3^
Δρ_min_ = −0.14 e Å^−3^

### 

Data collection: *CrystalClear* (Rigaku, 2001 ▶); cell refinement: *CrystalClear*; data reduction: *CrystalClear*; program(s) used to solve structure: *SHELXTL* (Sheldrick, 2008 ▶); program(s) used to refine structure: *SHELXTL*; molecular graphics: *ORTEP-3 for Windows* (Farrugia, 1997 ▶); software used to prepare material for publication: *SHELXTL*.

## Supplementary Material

Crystal structure: contains datablocks global, I. DOI: 10.1107/S1600536808017212/sg2245sup1.cif

Structure factors: contains datablocks I. DOI: 10.1107/S1600536808017212/sg2245Isup2.hkl

Additional supplementary materials:  crystallographic information; 3D view; checkCIF report

## Figures and Tables

**Table 1:** Selected bond lengths (Å)

O1—C6	1.227 (2)
O2—C12	1.222 (2)

**Fig. 1.**
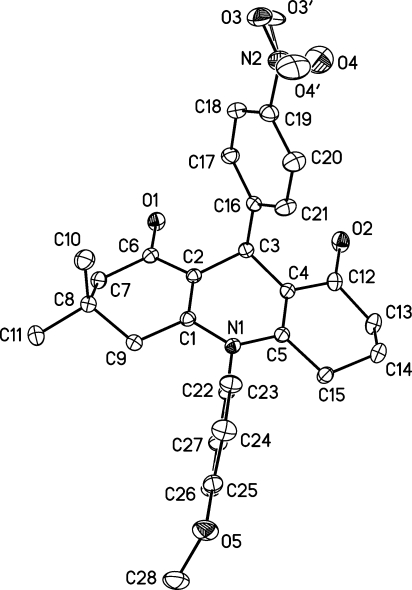
The molecular structure of (I), showing 20% probability displacement ellipsoids.

**Fig. 2.**
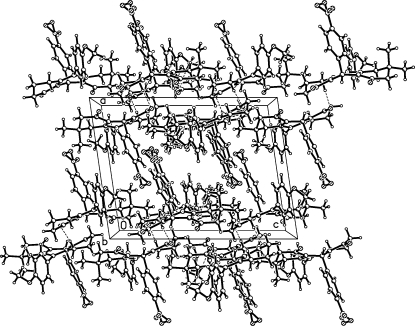
The molecular packing diagram in the crystal for (I).

**Fig. 3.**
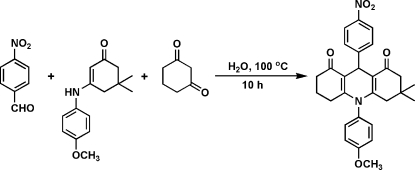
The formation of the title compound.

## supplementary crystallographic information

### Comment

With a 1,4-DHP parent nucleus, acridine-1,8-diones have been shown to have very high lasing efficiencies and have been used as photoinitiators (Shanmugasundaram, *et al.*, 1996).  Symmetrical acridinediones which contain two identical cyclohexanone rings fused to the DHP rings have been reported (Ganesh, *et al.*, 1998; Jang, *et al.*, 2005; Wang, *et al.*, 2003). However, the structures of acridinediones whose dihydropyridine ring linking two unsymmetrical cyclohexanone rings are rare.  Here we report the structure of unsymmetrical acridinedione(I). It was synthesized by the reaction of 4-nitrobenzaldehydes, 3-(4-Methoxy-phenylamino)-5,5-dimethyl-cyclohex-2-enone and 1,3-cyclohexanedione in refluxing water.

The acridine moiety (Figure 1) is nearly coplanar; the dihedral angle between the aromatic ring which is linked to the carbon and pyridine ring is 81.31°; the dihedral angle between the aromatic ring linked to the nitrogen and pyridine ring is 89.57°. The packing arrangement in a unit cell of the title molecule is shown in Fig. 2.

### Experimental

3,3-dimethyl-9-(4-nitrophenyl)-10-(4-methoxylphenyl)-decahydroacridine -1,8-dione were dissolved in ethanol. The mixture was set aside to crystallize. Suitable crystals for single-crystal X-ray diffraction were selected directly from the sample as prepared.

### Refinement

The H atoms bonded to N atom were located from difference density maps and refined isotropically. The H atoms bonded to C atoms were located geometrically and treated as riding, with C—H distances of 0.95–1.00 Å and with *U*_iso_(H) = 1.5Ueq(C) for methyl H atoms and 1.2Ueq(C) for others.

### Crystal data

**Table:**

C_28_H_28_N_2_O_5_	*F*_000_ = 1000
*M**_r_* = 472.52	*D*_x_ = 1.281 Mg m^−^^3^
Monoclinic, *P*2_1_/*c*	Melting point: 498 K
Hall symbol: -P 2ybc	Mo *K*α radiation λ = 0.71070 Å
*a* = 12.463 (2) Å	Cell parameters from 9180 reflections
*b* = 12.104 (2) Å	θ = 3.0–25.3º
*c* = 16.408 (3) Å	µ = 0.09 mm^−^^1^
β = 98.251 (5)º	*T* = 293 (2) K
*V* = 2449.6 (7) Å^3^	Block, yellow
*Z* = 4	0.80 × 0.59 × 0.58 mm

### Data collection

**Table:**

Rigaku Mercury diffractometer	4466 independent reflections
Radiation source: fine-focus sealed tube	3901 reflections with *I* > 2σ(*I*)
Monochromator: graphite	*R*_int_ = 0.030
Detector resolution: 7.31 pixels mm^-1^	θ_max_ = 25.4º
*T* = 293(2) K	θ_min_ = 3.0º
ω scans	*h* = −15→15
Absorption correction: multi-scan(CrystalClear; Rigaku, 2001)	*k* = −14→14
*T*_min_ = 0.760, *T*_max_ = 0.950	*l* = −19→19
23202 measured reflections	

### Refinement

**Table:**

Refinement on *F*^2^	Secondary atom site location: difference Fourier map
Least-squares matrix: full	Hydrogen site location: inferred from neighbouring sites
*R*[*F*^2^ > 2σ(*F*^2^)] = 0.054	H-atom parameters constrained
*wR*(*F*^2^) = 0.123	*w* = 1/[σ^2^(*F*_o_^2^) + (0.0452*P*)^2^ + 0.665*P*] where *P* = (*F*_o_^2^ + 2*F*_c_^2^)/3
*S* = 1.17	(Δ/σ)_max_ < 0.001
4466 reflections	Δρ_max_ = 0.15 e Å^−^^3^
338 parameters	Δρ_min_ = −0.14 e Å^−^^3^
Primary atom site location: structure-invariant direct methods	Extinction correction: none

### Special details

**Table:**

Geometry. All e.s.d.'s (except the e.s.d. in the dihedral angle between two l.s. planes) are estimated using the full covariance matrix. The cell e.s.d.'s are taken into account individually in the estimation of e.s.d.'s in distances, angles and torsion angles; correlations between e.s.d.'s in cell parameters are only used when they are defined by crystal symmetry. An approximate (isotropic) treatment of cell e.s.d.'s is used for estimating e.s.d.'s involving l.s. planes.
Refinement. Refinement of *F*^2^ against ALL reflections. The weighted *R*-factor *wR* and goodness of fit *S* are based on *F*^2^, conventional *R*-factors *R* are based on *F*, with *F* set to zero for negative *F*^2^. The threshold expression of *F*^2^ > σ(*F*^2^) is used only for calculating *R*-factors(gt) *etc*. and is not relevant to the choice of reflections for refinement. *R*-factors based on *F*^2^ are statistically about twice as large as those based on *F*, and *R*- factors based on ALL data will be even larger.

### Fractional atomic coordinates and isotropic or equivalent isotropic displacement parameters (Å^2^)

**Table:**

	*x*	*y*	*z*	*U*_iso_*/*U*_eq_	Occ. (<1)
O1	0.08638 (11)	1.24134 (11)	0.47495 (8)	0.0523 (4)	
O2	0.05773 (12)	1.11707 (12)	0.18637 (8)	0.0618 (4)	
O3	0.5690 (14)	1.4049 (12)	0.3168 (11)	0.082 (3)	0.50
O3'	0.5542 (16)	1.4082 (13)	0.3017 (12)	0.109 (5)	0.50
O4	0.6106 (9)	1.2617 (11)	0.2566 (5)	0.114 (3)	0.50
O4'	0.6318 (8)	1.2452 (10)	0.2980 (5)	0.102 (3)	0.50
O5	0.35763 (13)	0.47297 (12)	0.51861 (10)	0.0698 (4)	
N1	0.19057 (12)	0.88250 (11)	0.40821 (8)	0.0387 (3)	
N2	0.55282 (17)	1.3088 (2)	0.30009 (14)	0.0733 (6)	
C1	0.18027 (13)	0.96528 (14)	0.46587 (10)	0.0366 (4)	
C2	0.15262 (13)	1.06903 (13)	0.44043 (10)	0.0350 (4)	
C3	0.15223 (13)	1.10285 (13)	0.35148 (10)	0.0364 (4)	
H3	0.0951	1.1582	0.3377	0.044*	
C4	0.12365 (13)	1.00353 (14)	0.29784 (10)	0.0362 (4)	
C5	0.14825 (13)	0.90001 (14)	0.32513 (10)	0.0362 (4)	
C6	0.11987 (13)	1.14962 (14)	0.49803 (10)	0.0381 (4)	
C7	0.12427 (16)	1.11571 (16)	0.58669 (11)	0.0482 (5)	
H7A	0.0543	1.0859	0.5947	0.058*	
H7B	0.1375	1.1807	0.6212	0.058*	
C8	0.21130 (16)	1.03015 (15)	0.61444 (11)	0.0457 (4)	
C9	0.19433 (16)	0.93206 (14)	0.55494 (11)	0.0459 (4)	
H9A	0.2562	0.8829	0.5659	0.055*	
H9B	0.1307	0.8913	0.5653	0.055*	
C10	0.32415 (18)	1.08004 (19)	0.61482 (14)	0.0644 (6)	
H10A	0.3324	1.1032	0.5601	0.097*	
H10B	0.3782	1.0256	0.6335	0.097*	
H10C	0.3327	1.1426	0.6511	0.097*	
C11	0.2002 (2)	0.98929 (19)	0.70144 (12)	0.0662 (6)	
H11A	0.2555	0.9356	0.7186	0.099*	
H11B	0.1301	0.9562	0.7012	0.099*	
H11C	0.2079	1.0506	0.7390	0.099*	
C12	0.07097 (14)	1.02312 (16)	0.21323 (11)	0.0434 (4)	
C13	0.03387 (17)	0.92376 (17)	0.16216 (12)	0.0556 (5)	
H13A	0.0273	0.9427	0.1042	0.067*	
H13B	−0.0369	0.9009	0.1739	0.067*	
C14	0.11349 (17)	0.82947 (16)	0.18061 (11)	0.0516 (5)	
H14A	0.0867	0.7653	0.1486	0.062*	
H14B	0.1825	0.8503	0.1643	0.062*	
C15	0.13000 (16)	0.79997 (15)	0.27115 (11)	0.0458 (4)	
H15A	0.0667	0.7606	0.2840	0.055*	
H15B	0.1920	0.7511	0.2827	0.055*	
C16	0.25980 (13)	1.15546 (14)	0.33919 (10)	0.0366 (4)	
C17	0.28131 (16)	1.26474 (15)	0.36177 (12)	0.0490 (5)	
H17	0.2297	1.3049	0.3850	0.059*	
C18	0.37723 (17)	1.31493 (17)	0.35050 (12)	0.0549 (5)	
H18	0.3907	1.3881	0.3659	0.066*	
C19	0.45231 (15)	1.25482 (17)	0.31624 (12)	0.0495 (5)	
C20	0.43494 (16)	1.14667 (18)	0.29390 (14)	0.0603 (6)	
H20	0.4872	1.1070	0.2711	0.072*	
C21	0.33872 (15)	1.09742 (16)	0.30581 (13)	0.0522 (5)	
H21	0.3266	1.0238	0.2911	0.063*	
C22	0.23073 (14)	0.77423 (14)	0.43564 (10)	0.0391 (4)	
C23	0.34019 (15)	0.75163 (16)	0.44032 (13)	0.0521 (5)	
H23	0.3870	0.8052	0.4250	0.062*	
C24	0.38016 (17)	0.64987 (17)	0.46765 (14)	0.0596 (5)	
H24	0.4537	0.6346	0.4704	0.072*	
C25	0.31091 (17)	0.57056 (15)	0.49100 (12)	0.0494 (5)	
C26	0.20155 (16)	0.59242 (15)	0.48461 (12)	0.0492 (5)	
H26	0.1545	0.5385	0.4989	0.059*	
C27	0.16152 (15)	0.69446 (15)	0.45690 (11)	0.0451 (4)	
H27	0.0877	0.7090	0.4527	0.054*	
C28	0.2998 (2)	0.40427 (18)	0.56706 (16)	0.0784 (7)	
H28A	0.2784	0.4465	0.6116	0.118*	
H28B	0.3453	0.3441	0.5889	0.118*	
H28C	0.2364	0.3756	0.5335	0.118*	

### Atomic displacement parameters (Å^2^)

**Table:**

	*U*^11^	*U*^22^	*U*^33^	*U*^12^	*U*^13^	*U*^23^
O1	0.0602 (9)	0.0436 (8)	0.0537 (8)	0.0140 (6)	0.0105 (6)	−0.0031 (6)
O2	0.0759 (10)	0.0588 (9)	0.0479 (8)	0.0180 (8)	−0.0002 (7)	0.0060 (7)
O3	0.078 (5)	0.076 (6)	0.096 (5)	−0.035 (3)	0.028 (5)	−0.016 (5)
O3'	0.096 (6)	0.084 (6)	0.145 (10)	−0.049 (4)	0.009 (5)	0.041 (6)
O4	0.075 (6)	0.145 (8)	0.135 (7)	−0.039 (5)	0.057 (5)	−0.039 (6)
O4'	0.048 (3)	0.103 (4)	0.160 (8)	−0.012 (3)	0.034 (5)	−0.008 (6)
O5	0.0831 (11)	0.0435 (8)	0.0836 (11)	0.0198 (7)	0.0153 (8)	0.0137 (7)
N1	0.0463 (8)	0.0316 (7)	0.0379 (8)	0.0016 (6)	0.0053 (6)	−0.0016 (6)
N2	0.0583 (13)	0.0830 (16)	0.0799 (15)	−0.0259 (12)	0.0148 (11)	−0.0027 (14)
C1	0.0348 (9)	0.0364 (9)	0.0390 (9)	−0.0037 (7)	0.0064 (7)	−0.0027 (7)
C2	0.0334 (9)	0.0337 (9)	0.0383 (9)	−0.0013 (7)	0.0069 (7)	−0.0016 (7)
C3	0.0371 (9)	0.0342 (9)	0.0378 (9)	0.0039 (7)	0.0056 (7)	0.0010 (7)
C4	0.0324 (9)	0.0389 (9)	0.0377 (9)	−0.0005 (7)	0.0066 (7)	−0.0027 (7)
C5	0.0322 (9)	0.0386 (9)	0.0386 (9)	−0.0033 (7)	0.0080 (7)	−0.0030 (7)
C6	0.0325 (9)	0.0383 (10)	0.0440 (10)	−0.0012 (7)	0.0076 (7)	−0.0039 (8)
C7	0.0569 (12)	0.0462 (11)	0.0437 (10)	−0.0012 (9)	0.0146 (9)	−0.0062 (8)
C8	0.0562 (12)	0.0423 (10)	0.0385 (10)	−0.0048 (9)	0.0068 (8)	−0.0017 (8)
C9	0.0589 (12)	0.0393 (10)	0.0396 (10)	−0.0030 (9)	0.0074 (8)	0.0020 (8)
C10	0.0601 (14)	0.0671 (14)	0.0610 (13)	−0.0104 (11)	−0.0076 (10)	0.0018 (11)
C11	0.0981 (18)	0.0610 (14)	0.0400 (11)	−0.0044 (12)	0.0111 (11)	0.0001 (10)
C12	0.0374 (10)	0.0524 (12)	0.0405 (10)	0.0068 (8)	0.0064 (7)	−0.0030 (9)
C13	0.0523 (12)	0.0662 (13)	0.0455 (11)	0.0043 (10)	−0.0018 (9)	−0.0136 (10)
C14	0.0555 (12)	0.0541 (12)	0.0448 (11)	0.0013 (9)	0.0054 (9)	−0.0145 (9)
C15	0.0503 (11)	0.0414 (10)	0.0467 (10)	−0.0052 (8)	0.0101 (8)	−0.0090 (8)
C16	0.0395 (9)	0.0355 (9)	0.0347 (9)	0.0005 (7)	0.0048 (7)	0.0029 (7)
C17	0.0558 (12)	0.0393 (10)	0.0548 (11)	−0.0031 (9)	0.0180 (9)	−0.0036 (9)
C18	0.0645 (13)	0.0429 (11)	0.0582 (12)	−0.0142 (10)	0.0117 (10)	−0.0026 (9)
C19	0.0422 (11)	0.0553 (12)	0.0508 (11)	−0.0114 (9)	0.0056 (8)	0.0048 (9)
C20	0.0425 (11)	0.0588 (13)	0.0823 (15)	−0.0017 (10)	0.0182 (10)	−0.0091 (11)
C21	0.0442 (11)	0.0406 (10)	0.0733 (14)	−0.0018 (8)	0.0136 (9)	−0.0070 (9)
C22	0.0444 (10)	0.0322 (9)	0.0411 (9)	0.0028 (7)	0.0070 (7)	−0.0017 (7)
C23	0.0442 (11)	0.0462 (11)	0.0673 (13)	−0.0008 (8)	0.0133 (9)	0.0081 (9)
C24	0.0461 (12)	0.0547 (12)	0.0794 (15)	0.0121 (10)	0.0134 (10)	0.0090 (11)
C25	0.0606 (13)	0.0361 (10)	0.0515 (11)	0.0096 (9)	0.0086 (9)	0.0006 (8)
C26	0.0571 (12)	0.0368 (10)	0.0551 (12)	−0.0048 (9)	0.0124 (9)	0.0020 (8)
C27	0.0440 (10)	0.0398 (10)	0.0521 (11)	0.0011 (8)	0.0093 (8)	0.0007 (8)
C28	0.103 (2)	0.0432 (12)	0.0852 (17)	0.0004 (12)	0.0012 (14)	0.0173 (12)

### Geometric parameters (Å, °)

**Table:**

O1—C6	1.227 (2)	C11—H11B	0.9600
O2—C12	1.222 (2)	C11—H11C	0.9600
O3—N2	1.206 (13)	C12—C13	1.500 (3)
O3'—N2	1.204 (16)	C13—C14	1.514 (3)
O4—N2	1.225 (12)	C13—H13A	0.9700
O4'—N2	1.254 (11)	C13—H13B	0.9700
O5—C25	1.365 (2)	C14—C15	1.513 (3)
O5—C28	1.417 (3)	C14—H14A	0.9700
N1—C1	1.396 (2)	C14—H14B	0.9700
N1—C5	1.405 (2)	C15—H15A	0.9700
N1—C22	1.451 (2)	C15—H15B	0.9700
N2—C19	1.470 (3)	C16—C21	1.384 (2)
C1—C2	1.352 (2)	C16—C17	1.389 (2)
C1—C9	1.502 (2)	C17—C18	1.377 (3)
C2—C6	1.457 (2)	C17—H17	0.9300
C2—C3	1.515 (2)	C18—C19	1.368 (3)
C3—C4	1.502 (2)	C18—H18	0.9300
C3—C16	1.524 (2)	C19—C20	1.368 (3)
C3—H3	0.9800	C20—C21	1.378 (3)
C4—C5	1.351 (2)	C20—H20	0.9300
C4—C12	1.467 (2)	C21—H21	0.9300
C5—C15	1.498 (2)	C22—C27	1.373 (2)
C6—C7	1.505 (3)	C22—C23	1.383 (3)
C7—C8	1.521 (3)	C23—C24	1.379 (3)
C7—H7A	0.9700	C23—H23	0.9300
C7—H7B	0.9700	C24—C25	1.381 (3)
C8—C10	1.530 (3)	C24—H24	0.9300
C8—C9	1.532 (2)	C25—C26	1.378 (3)
C8—C11	1.536 (3)	C26—C27	1.384 (3)
C9—H9A	0.9700	C26—H26	0.9300
C9—H9B	0.9700	C27—H27	0.9300
C10—H10A	0.9600	C28—H28A	0.9600
C10—H10B	0.9600	C28—H28B	0.9600
C10—H10C	0.9600	C28—H28C	0.9600
C11—H11A	0.9600		
			
C25—O5—C28	117.82 (18)	H11B—C11—H11C	109.5
C1—N1—C5	119.39 (14)	O2—C12—C4	120.70 (17)
C1—N1—C22	119.93 (14)	O2—C12—C13	121.97 (17)
C5—N1—C22	120.11 (13)	C4—C12—C13	117.33 (16)
O3'—N2—O3	14 (2)	C12—C13—C14	110.60 (16)
O3'—N2—O4	118.0 (9)	C12—C13—H13A	109.5
O3—N2—O4	119.3 (9)	C14—C13—H13A	109.5
O3'—N2—O4'	127.3 (10)	C12—C13—H13B	109.5
O3—N2—O4'	119.5 (10)	C14—C13—H13B	109.5
O4—N2—O4'	33.8 (5)	H13A—C13—H13B	108.1
O3'—N2—C19	116.8 (8)	C15—C14—C13	111.54 (16)
O3—N2—C19	120.4 (7)	C15—C14—H14A	109.3
O4—N2—C19	119.1 (6)	C13—C14—H14A	109.3
O4'—N2—C19	115.1 (5)	C15—C14—H14B	109.3
C2—C1—N1	120.08 (15)	C13—C14—H14B	109.3
C2—C1—C9	122.55 (15)	H14A—C14—H14B	108.0
N1—C1—C9	117.26 (14)	C5—C15—C14	112.17 (15)
C1—C2—C6	120.29 (15)	C5—C15—H15A	109.2
C1—C2—C3	120.91 (15)	C14—C15—H15A	109.2
C6—C2—C3	118.78 (14)	C5—C15—H15B	109.2
C4—C3—C2	108.41 (13)	C14—C15—H15B	109.2
C4—C3—C16	113.33 (13)	H15A—C15—H15B	107.9
C2—C3—C16	111.05 (13)	C21—C16—C17	117.90 (17)
C4—C3—H3	108.0	C21—C16—C3	121.94 (15)
C2—C3—H3	108.0	C17—C16—C3	120.16 (15)
C16—C3—H3	108.0	C18—C17—C16	121.48 (18)
C5—C4—C12	120.85 (16)	C18—C17—H17	119.3
C5—C4—C3	121.65 (15)	C16—C17—H17	119.3
C12—C4—C3	117.47 (15)	C19—C18—C17	118.61 (18)
C4—C5—N1	119.80 (15)	C19—C18—H18	120.7
C4—C5—C15	123.08 (16)	C17—C18—H18	120.7
N1—C5—C15	117.10 (15)	C18—C19—C20	121.84 (18)
O1—C6—C2	121.36 (16)	C18—C19—N2	119.24 (19)
O1—C6—C7	120.58 (15)	C20—C19—N2	118.88 (19)
C2—C6—C7	118.02 (15)	C19—C20—C21	118.90 (19)
C6—C7—C8	113.60 (15)	C19—C20—H20	120.5
C6—C7—H7A	108.8	C21—C20—H20	120.5
C8—C7—H7A	108.8	C20—C21—C16	121.26 (18)
C6—C7—H7B	108.8	C20—C21—H21	119.4
C8—C7—H7B	108.8	C16—C21—H21	119.4
H7A—C7—H7B	107.7	C27—C22—C23	119.85 (16)
C7—C8—C10	110.37 (16)	C27—C22—N1	120.84 (16)
C7—C8—C9	108.03 (15)	C23—C22—N1	119.31 (16)
C10—C8—C9	110.56 (16)	C24—C23—C22	120.14 (18)
C7—C8—C11	109.98 (16)	C24—C23—H23	119.9
C10—C8—C11	109.21 (17)	C22—C23—H23	119.9
C9—C8—C11	108.67 (15)	C23—C24—C25	119.99 (19)
C1—C9—C8	113.50 (15)	C23—C24—H24	120.0
C1—C9—H9A	108.9	C25—C24—H24	120.0
C8—C9—H9A	108.9	O5—C25—C26	124.17 (18)
C1—C9—H9B	108.9	O5—C25—C24	116.08 (18)
C8—C9—H9B	108.9	C26—C25—C24	119.75 (17)
H9A—C9—H9B	107.7	C25—C26—C27	120.16 (18)
C8—C10—H10A	109.5	C25—C26—H26	119.9
C8—C10—H10B	109.5	C27—C26—H26	119.9
H10A—C10—H10B	109.5	C22—C27—C26	120.07 (17)
C8—C10—H10C	109.5	C22—C27—H27	120.0
H10A—C10—H10C	109.5	C26—C27—H27	120.0
H10B—C10—H10C	109.5	O5—C28—H28A	109.5
C8—C11—H11A	109.5	O5—C28—H28B	109.5
C8—C11—H11B	109.5	H28A—C28—H28B	109.5
H11A—C11—H11B	109.5	O5—C28—H28C	109.5
C8—C11—H11C	109.5	H28A—C28—H28C	109.5
H11A—C11—H11C	109.5	H28B—C28—H28C	109.5
			
C5—N1—C1—C2	−14.8 (2)	C4—C12—C13—C14	−37.0 (2)
C22—N1—C1—C2	173.82 (15)	C12—C13—C14—C15	56.9 (2)
C5—N1—C1—C9	161.57 (15)	C4—C5—C15—C14	16.8 (2)
C22—N1—C1—C9	−9.8 (2)	N1—C5—C15—C14	−164.75 (15)
N1—C1—C2—C6	167.22 (15)	C13—C14—C15—C5	−46.8 (2)
C9—C1—C2—C6	−8.9 (3)	C4—C3—C16—C21	−21.4 (2)
N1—C1—C2—C3	−11.2 (2)	C2—C3—C16—C21	100.90 (19)
C9—C1—C2—C3	172.67 (15)	C4—C3—C16—C17	158.68 (16)
C1—C2—C3—C4	31.2 (2)	C2—C3—C16—C17	−79.0 (2)
C6—C2—C3—C4	−147.19 (15)	C21—C16—C17—C18	0.9 (3)
C1—C2—C3—C16	−93.89 (18)	C3—C16—C17—C18	−179.21 (17)
C6—C2—C3—C16	87.68 (17)	C16—C17—C18—C19	0.0 (3)
C2—C3—C4—C5	−29.3 (2)	C17—C18—C19—C20	−0.7 (3)
C16—C3—C4—C5	94.51 (18)	C17—C18—C19—N2	177.03 (19)
C2—C3—C4—C12	152.76 (14)	O3'—N2—C19—C18	−15.2 (12)
C16—C3—C4—C12	−83.47 (18)	O3—N2—C19—C18	0.0 (12)
C12—C4—C5—N1	−174.83 (14)	O4—N2—C19—C18	−167.1 (5)
C3—C4—C5—N1	7.3 (2)	O4'—N2—C19—C18	155.0 (5)
C12—C4—C5—C15	3.5 (3)	O3'—N2—C19—C20	162.6 (11)
C3—C4—C5—C15	−174.38 (15)	O3—N2—C19—C20	177.8 (11)
C1—N1—C5—C4	16.9 (2)	O4—N2—C19—C20	10.7 (6)
C22—N1—C5—C4	−171.72 (15)	O4'—N2—C19—C20	−27.2 (5)
C1—N1—C5—C15	−161.57 (15)	C18—C19—C20—C21	0.5 (3)
C22—N1—C5—C15	9.8 (2)	N2—C19—C20—C21	−177.3 (2)
C1—C2—C6—O1	−174.22 (16)	C19—C20—C21—C16	0.5 (3)
C3—C2—C6—O1	4.2 (2)	C17—C16—C21—C20	−1.1 (3)
C1—C2—C6—C7	3.5 (2)	C3—C16—C21—C20	178.99 (18)
C3—C2—C6—C7	−178.04 (15)	C1—N1—C22—C27	86.5 (2)
O1—C6—C7—C8	−152.72 (17)	C5—N1—C22—C27	−84.8 (2)
C2—C6—C7—C8	29.5 (2)	C1—N1—C22—C23	−93.5 (2)
C6—C7—C8—C10	66.9 (2)	C5—N1—C22—C23	95.2 (2)
C6—C7—C8—C9	−54.1 (2)	C27—C22—C23—C24	−1.1 (3)
C6—C7—C8—C11	−172.55 (16)	N1—C22—C23—C24	178.85 (18)
C2—C1—C9—C8	−18.8 (2)	C22—C23—C24—C25	−0.5 (3)
N1—C1—C9—C8	164.92 (15)	C28—O5—C25—C26	−20.7 (3)
C7—C8—C9—C1	48.7 (2)	C28—O5—C25—C24	160.1 (2)
C10—C8—C9—C1	−72.2 (2)	C23—C24—C25—O5	−178.88 (19)
C11—C8—C9—C1	168.00 (17)	C23—C24—C25—C26	1.9 (3)
C5—C4—C12—O2	−173.30 (17)	O5—C25—C26—C27	179.16 (18)
C3—C4—C12—O2	4.7 (2)	C24—C25—C26—C27	−1.7 (3)
C5—C4—C12—C13	7.1 (2)	C23—C22—C27—C26	1.4 (3)
C3—C4—C12—C13	−174.85 (15)	N1—C22—C27—C26	−178.62 (16)
O2—C12—C13—C14	143.43 (19)	C25—C26—C27—C22	0.0 (3)
